# Large net forest loss in Cambodia’s Tonle Sap Lake protected areas during 1992–2019

**DOI:** 10.1007/s13280-022-01704-4

**Published:** 2022-02-08

**Authors:** Aifang Chen, Anping Chen, Olli Varis, Deliang Chen

**Affiliations:** 1 School of Environmental Science and Engineering, Southern University of Science and Technology, Shenzhen, 518055 China; 2 Regional Climate Group, Department of Earth Sciences, University of Gothenburg, Box 460, 40530 Gothenburg, Sweden; 3 Department of Biology and Graduate Degree Program in Ecology, Colorado State University, Fort Collins, CO 80523 USA; 4 Water and Development Research Group, Aalto University, 15200 Espoo, Finland

**Keywords:** Cropland expansion, Forest loss, Land-use/land cover change, Mekong, Tonle Sap Lake

## Abstract

Historical land-use practices have caused forest loss in Cambodia’s Tonle Sap Lake area (TSLA), the largest freshwater lake in Southeast Asia. However, it remains unclear if this deforestation trend had continued since 2001 when the land was designated as protected areas. Using satellite imagery, we investigated forest conversion flows and fragmentation patterns in the TSLA for 1992–2001, 2001–2010, and 2010–2019, respectively. Results show substantial forest losses and fragmentations occurring at the lower floodplain where the protected areas are located until 2010, with some forest regain during 2010–2019. The land conversions indicated that forest clearing and agricultural farming were the primary causes for observed extensive forest loss during 1992–2010. Hence, despite the creating of protected areas in 2001, our findings reveal the persistence of alarming forest loss in the TSLA until 2010. On the other hand, while net forest loss has stopped after 2010, forest regain during 2010–2019 is way too small to restore the region’s total forest area to even the level when the protected areas were established. Thus, more effective planning and implementations of forest management and restoration policies are needed for the TSLA.

**Supplementary Information:**

The online version contains supplementary material available at 10.1007/s13280-022-01704-4.

## Introduction

Forests provide essential ecosystem services including mitigating greenhouse gas emissions, regulating water flow, supporting aquatic ecosystem and freshwater fisheries, maintaining soil fertility, sustaining wildlife and biodiversity, and providing a source of income for residents (Keskinen 2003; Arias et al. 2012; MOWRAM et al. 2013). Globally, however, forests have been shrinking over the last decades, threatening these essential natural services provided by forest ecosystems (Foley et al. 2005; Hansen et al. 2013; Haddad et al. 2015; Curtis et al. 2018). In particular, tropical deforestation has been a key source of man-made carbon emission, profoundly contributing to global biodiversity loss and changing the quantity and quality of ecosystem services it provides because of the vital role of the tropical forest in the Earth system dynamics (Foley et al. 2005; Alroy 2017). Among the world’s major tropical regions, Southeast Asia has witnessed one of the fastest deforestation rates in recent decades (Hansen et al. 2013; Dong et al. 2014; Zeng et al. 2018; Potapov et al. 2019). The increasing regional and international demands for food and logs, and urbanization, among others, have been suggested as major drivers of recent fast deforestation in tropical Southeast Asia (Fox et al. 2014; Davis et al. 2015; Curtis et al. 2018). Given the important ecosystem services provided by tropical forests, it is critical to quantify the spatial pattern and possible drivers of recent forest changes in Southeast Asia, particularly for some key ecoregions such as the Tonle Sap Lake area (TSLA) in Cambodia.

**Fig. 1:**
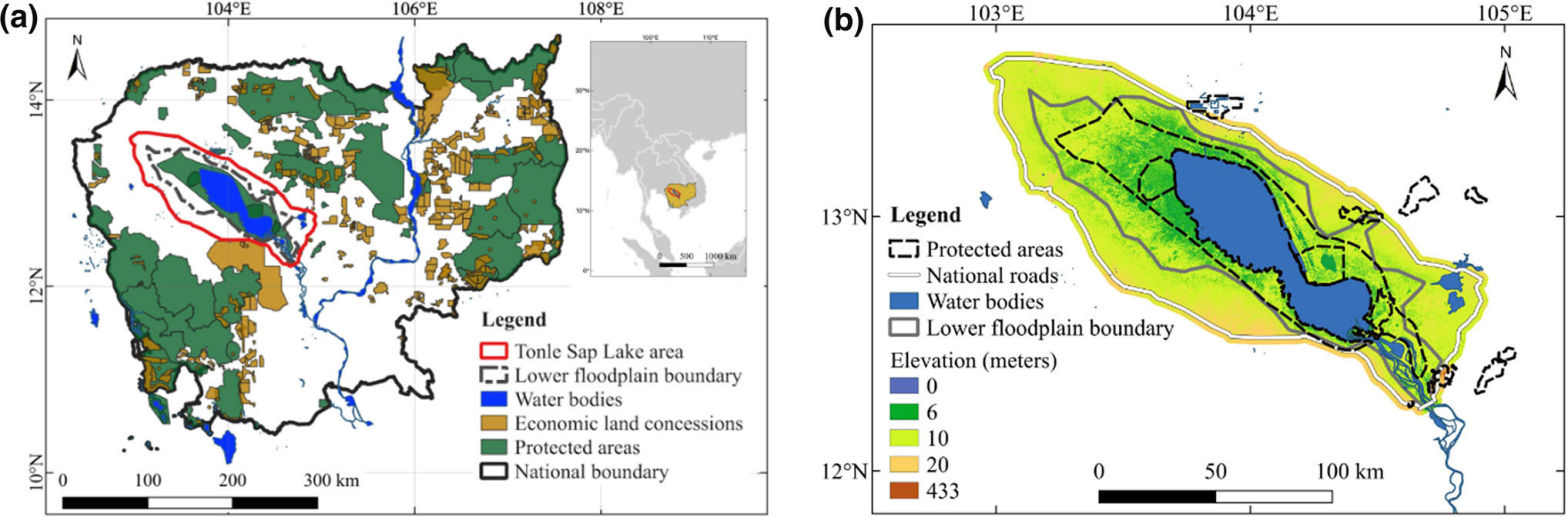
Cambodia and the Tonle Sap Lake area. **a** The solid red line is the boundary of the Tonle Sap Lake area, and the dash gray line is the boundary of the lower floodplain. **b** Tonle Sap Lake area comprises the lake-floodplain area between National Roads 5 and 6 and an additional 3 km farther from the National Roads. Natural protected areas and economic land concessions in Cambodia are obtained from the Open Development Cambodia (https://opendevelopmentcambodia.net/)

Tonle Sap Lake is a constitutive element of the Mekong River system in Mainland Southeast Asia and an exceptional lake-floodplain system (Salmivaara et al. 2016; Uk et al. 2018). Connecting to the Mekong River and driven by the summer monsoon, the lake’s water surface fluctuates in an annual cycle from ~ 2500 km^2^ in the dry season to ~ 15 000 km^2^ in the wet season, creating a so-called annual monomodal flood pulse (Junk et al. 1989; ADB 2005; Kummu and Sarkkula 2008). Such flood pulse drives seasonally inundated floodplains, cultivating notably productive inland wetlands in Southeast Asia, and providing refuges for many endangered species (Campbell et al. 2006; Lamberts 2006; Ziv et al. 2012). In addition, Tonle Sap Lake offers various ecosystem services and economic values and fosters unique cultures (Keskinen 2003; Kummu et al. 2006; Chadwick et al. 2008; Uk et al. 2018), including the provision of water resources for domestic use, agriculture, aquaculture, transportation, modifying local climate (Chadwick et al. 2008), and acting as a natural reservoir for the downstream area of the Mekong River Basin (Kummu et al. 2006). More than 1.7 million people in Cambodia rely on the Tonle Sap Lake (Salmivaara et al. 2016). Hence, Tonle Sap Lake is fundamental for the local and regional environment, economy, and society (Bonheur and Lane 2002; Lamberts 2006; Uk et al. 2018). To reconcile biodiversity conservation with sustainable development, the TSLA has been designated as a UNESCO Biosphere Reserve since 2001 (Fig. 1a) (UNESCO Phnom Penh Office 2018) and maintained three Ramsar sites under the Convention on Wetlands (Sithirith 2015). The establishment of protected areas is a common policy tool for protecting forests and other ecosystems (Collins and Mitchard 2017).

Like many other areas of Southeast Asia, the TSLA has witnessed extensive land-use/land cover change (LUCC) since the 1960s, characterized by rapid deforestation and agricultural expansion (ADB 2005; MOWRAM et al. 2013). Forest cover in the Tonle Sap watershed has decreased by 43% over 1990–2009, even higher than Cambodia’s national average deforestation rate (Senevirathne et al. 2010; MOWRAM et al. 2013). Lohani et al. (2020) also found a higher forest loss rate in the Tonle Sap than in Cambodia over 1993–2017, even though the net forest loss was modest (about 1944 km^2^). At the same time, agricultural land expanded by 34%, making it one of the leading factors of the diminishing forests in the TSLA (Senevirathne et al. 2010; Song et al. 2011). Rising demands for agricultural products and job opportunities have brought major challenges for the region’s economy, which is heavily based on the aquatic agricultural system, and put additional pressure on the TSLA ecosystem (Salmivaara et al. 2016). Protected areas are usually expected to stop or even reverse forest loss. However, forest loss has still been found in some protected areas in the tropics, including Cambodian protected regions such as Snoul, Phnom Kulen, Beng Per, and Samlaut (Collins and Mitchard 2017).

The critical question is: as one of the most prominent protected areas in Cambodia and even the Mainland Southeast Asia, has the declaration of the TSLA as a key protected area in 2001 helped slow down, stop, or even reverse the deforestation in this region? Lohani et al. (2020) analyzed spatiotemporal patterns of deforestation in Cambodia and the neighboring 3S (Srepok, Sean, and Sekong) watershed and Tonle Sap region, based on the Landsat satellite image and a random forest model. Potapov et al. (2019) presented an approach for annual Landsat-based woody vegetation structure monitoring for the lower Mekong region for 2000–2017. While these studies are important, they are more on LUCC per se. In addition, Tonle Sap Lake offers various ecosystem services and economic values and fosters unique cultures, and its lower and upper floodplains are dominated by different land cover types with diverse socioeconomic importance. Therefore, studies showing patterns of forest cover change in each floodplain could help understand the forest loss in the Tonle Sap Lake protected area (Forest Trends 2015). This type of assessments, however, are still scarce, impeding the understanding of the efficiency of the protected area and the identification of potential issues of forest policy implementation.

Here, we assessed the LUCC with a particular focus on the forest cover change in the TSLA during 1992–2019, which was further equally divided into three sub-periods (1992–2001, 2001–2010, and 2010–2019), using the European Space Agency (ESA) Climate Change Initiative (CCI) Land Cover product. ESA CCI provides the first long-term and successive annual time series of land cover change for 1992–2019. It has been validated by external parties and applied in various high-impact societal issues, for instance, analyzing carbon and water budgets with land surface models (Bontemps et al. 2012; ESA 2017; Li et al. 2018; Chen et al. 2020). By comparing the LUCC and forest cover change in the TSLA, we aimed to provide a better understanding on changes in forest area in the TSLA protected area, which is critical for informing policy decisions and economic values for ecosystem protection. We also analyzed forest cover change and other LUCC for the lower and upper floodplains separately, allowing for the analysis of interconnections between social-economic and management activities and land cover changes. Thus, our results provide insights for future forest policies and their implementations in Southeast Asia and other tropical regions facing similar challenges.

## Materials and methods

### Study area

The TSLA has a vast flat terrain, with water levels ranging between 1 and 9 m above mean sea level (m a.m.s.l.) during dry and wet seasons, respectively (Arias et al. 2012). The floodplain of the TSLA is a crucial region for agricultural production in Cambodia, extending to six provinces between Cambodian National Roads 5 and 6 (Kampong Chhnang, Pursat, Battambang, Banteay Meanchey, Siem Reap, and Kampong Thom) (Song et al. 2011). Following Salmivaara et al. (2016), the TSLA is defined as comprising the lake-floodplain area between Cambodian National Roads 5 and 6 and an additional 3 km farther from the National Roads (Fig. 1b). Thus, the TSLA floodplain comprises two subzones: the lower floodplain (area enclosed by the 6-m elevation contour [a.m.s.l.]) and the upper floodplain (area beyond the 6-m elevation contour to the 3-km buffer beyond the National Roads [> 10 m a.m.s.l.]), with a total surface area of 16,491 km^2^ (Keskinen 2006; Salmivaara et al. 2016). The lower floodplain is dominated by flooded forests, and the upper floodplain is mainly used for residential areas and crop cultivation.

### Land cover data

ESA CCI land cover product (http://www.esa-landcover-cci.org/) offers annual global land cover classifications with 300 m spatial resolution from 1992. It is developed with machine learning and unsupervised classifications based on the global daily surface reflectance observations from AVHRR, MERIS, PROBA-V, SPOT-VGT, Sentinel-3 OLCI, and SLSTR (ESA 2017). Among many land cover data products, ESA CCI is the first one to provide long-term consistent annual land cover maps defined by the commonly used Land Cover Classification System (LCCS) of the United Nations (UN) Food and Agriculture Organization (FAO). Assessed by external parties, the ESA CCI land-use product is proved to have an overall accuracy of 71%. Furthermore, on a global scale, it has a correlation coefficient of 0.68 with the United States Geological Survey (USGS) global land cover validation data (Chen et al. 2020). Besides, it has played an essential role in simulating the carbon dynamics and land cover change feedbacks on climate (Li et al. 2018). We used the ESA CCI land cover product to identify LUCC in the TSLA from 1992 to 2019. The product includes 22 major land cover types based on the LCCS classification. We then aggregated them in the TSLA and in Cambodia into seven land cover types, as shown in Table 1.

**Table 1:** Land cover types aggregation from 22 major land cover types of the LCCS classification

Aggregated land cover	Original land cover
Forests	Tree cover, broadleaved, evergreen, closed to open [> 15%]; tree cover, broadleaved, deciduous, closed to open [> 15%]; tree cover, needleleaved, evergreen, closed to open [> 15%]; tree cover, needleleaved, deciduous, closed to open [> 15%]; tree cover, mixed leaf type [broadleaved and needleleaved]; mosaic tree and shrub [> 50%] /herbaceous cover [< 50%]; tree cover, flooded, fresh or brackish water; tree cover, flooded, saline water in ESA CCI
Shrubs	Mosaic herbaceous cover [> 50%] /tree and shrub [< 50%]; shrubland; grassland
Croplands	Rainfed cropland; irrigated cropland; mosaic cropland [> 50%] /natural vegetation [tree, shrub, herbaceous cover] [< 50%]; mosaic natural vegetation [tree, shrub, herbaceous cover][> 50%] /cropland [< 50%]
Wetlands	Shrub or herbaceous cover; flooded; fresh-saline or brackish water
Urban	Urban
Water bodies	Water bodies
Others	Bare areas; sparse vegetation; lichens and mosses; and permanent snow and ice

### Methods

To estimate the annual forest cover change and evaluate the performance of the protected area policy in the Tonle Sap Lake protected area established since 2001 (Forest Trends 2015), we first conducted spatiotemporal analyses of forest fragmentation changes in the TSLA for 1992–2019 as follows: (a) As the study period was equally divided into three sub-periods (i.e., 1992–2001, 2001–2010, and 2010–2019), we demonstrated continuous forest loss until 2010 with land cover maps of the TSLA for the four years separating the three sub-periods (i.e., 1992, 2001, 2010, and 2019); (b) As forest fragmentation greatly impairs wildlife habitats and biodiversity (Riitters et al. 2000; Haddad et al. 2015) and threatens the status of the TSLA as a biodiversity hotspot, we then conducted a forest fragmentation analysis to reveal spatial patterns of forest fragmentation for the same four years; (c) We also presented the annual time series of land cover changes in the TSLA and in Cambodia, along with spatial pattern of land cover changes during 1992–2019, which would show the spatial processes of annual forest loss and cropland expansion and enable further investigations on their drivers such as the impacts of human forest management and agricultural activities.

Next, we conducted a land cover conversion analysis to reveal significant land cover transitions resulting from forest loss for 1992–2001, 2001–2010, and 2010–2019. Since the upper and lower floodplains have different social-economic activities (Salmivaara et al. 2016), we performed the land cover conversion analysis for the lower and upper floodplains, respectively, showing different land conversion flows driven by diverse human activities. Furthermore, the annual time series of forest loss and the associated land conversion flows in the whole TSLA during 1992–2019 were analyzed to reveal a more in-depth change of forests.

The ‘Fragmentation Model’ developed by Riitters et al. (2000, 2016) has been found useful for forest loss investigations (Li et al. 2010; Dong et al. 2014). Here, we conducted a forest fragmentation analysis by comparing the forest interior area for 1992, 2001, 2010, and 2019. Forest interior area is of high ecological importance because the non-interior forest is at high risk from ‘edge effects,’ including a high risk of invasive species (Riitters et al. 2016). The forest interior is defined by its forest area density (FAD). A ‘moving windows’ algorithm with fixed-area ‘windows’ (i.e., 5 × 5 pixels) was applied to assess the FAD, which is the number of forest pixels divided by the total number of pixels in the fixed-area ‘windows’ (Eq. [1]). The window was centered on each extant forest pixel, and FAD was measured for the window (Riitters et al. 2000). The central pixels were then labeled as the interior if the associated *P*_*f*_ ≥ 0.9. Since the fragmentation model is sensitive to the fixed-area ‘windows’ scale (Riitters et al. 2000; Dong et al. 2014), various sizes of ‘windows’ (such as 3 × 3 and 7 × 7 pixels) were used for sensitivity analysis. More details about the ‘Fragmentation Model’ can be found in Riitters et al. (2000, 2016).

1 $${P}_{\mathrm{f}}={N}_{\mathrm{f}}/{N}_{\mathrm{total}},$$

where *N*_f_ is the number of forest pixels in the fixed-area ‘windows’; *N*_total_ is the total number of pixels in the fixed-area ‘windows’ (which is 25 in the 5 × 5 pixels ‘windows’).

To analyze the potential driving force of forest loss in the TSLA, we used the 2009–2019/2020 regional statistics of agricultural, fishing, and forestry activities for the Tonle Sap region from the Cambodia Socio-Economic Survey, conducted by the National Institute of Statistics of Cambodia (www.nis.gov.kh) (NIS 2020a). Although the often referred Tonle Sap region in governmental socioeconomic statistics is more extensive than the TSLA, the TSLA is a major part of the Tonle Sap region. And Tonle Sap Lake is the largest fishery area in Cambodia and the Tonle Sap region (Salmivaara et al. 2016; Uk et al. 2018). Therefore, the statistics can reveal the agricultural, fishing, and forestry activities in the TSLA to some extent, and assist exploring the drivers of forest cover change in the TSLA. Moreover, we applied the non-parametric Mann–Kendall trend test for monotonic trends (Kendall 1938) with a confidence level of 95% (*p* < 0.05) and Sen's slope (Sen 1968) for measuring the trend magnitude (Lin and Qi 2017).

## Results

### Forest fragmentation in the TSLA

**Fig. 2:**
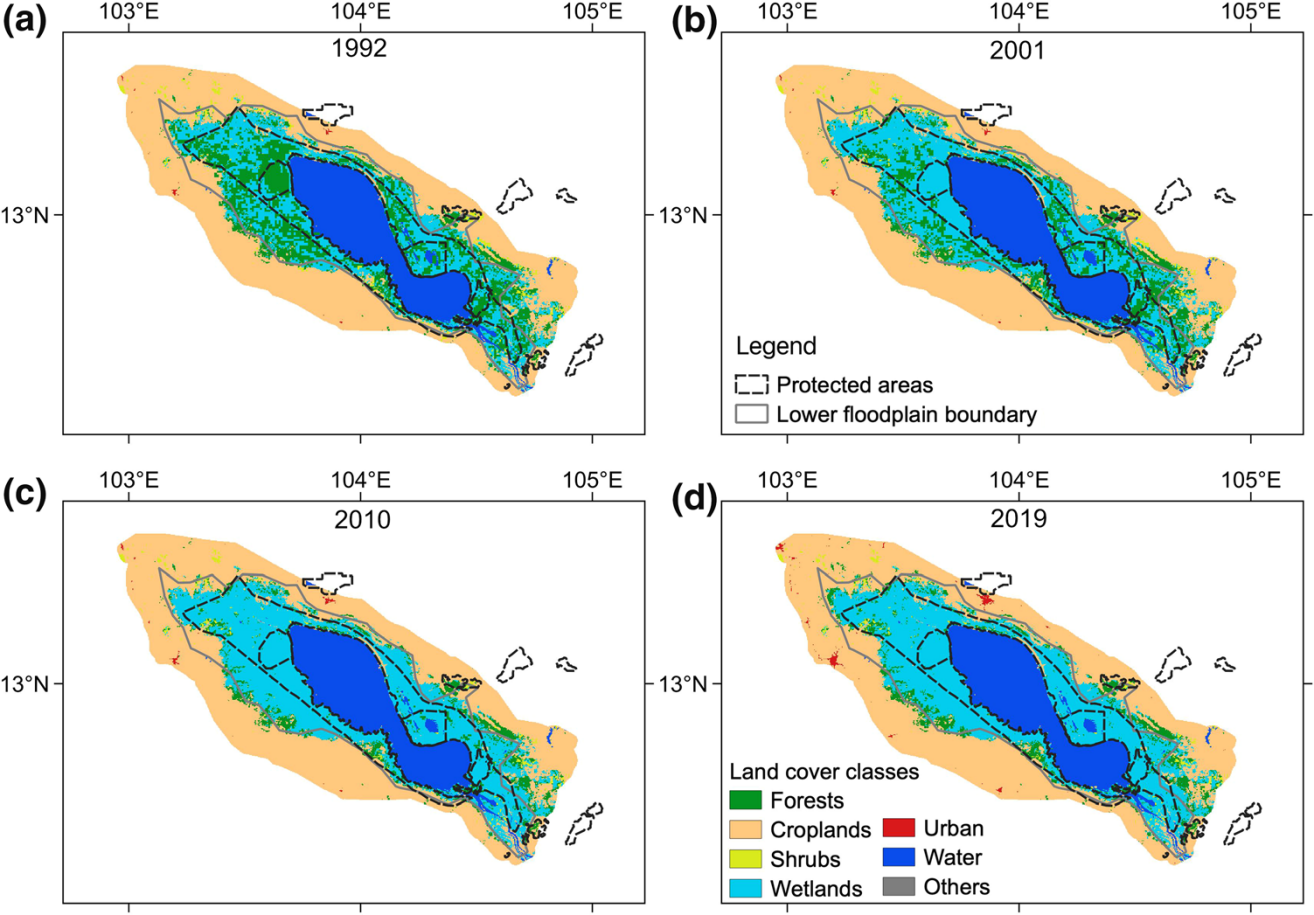
Spatial patterns of different land cover types in the Tonle Sap Lake area in **a** 1992, **b** 2001, **c** 2010, and **d** 2019

Figure 2 shows the spatial patterns of different land cover types for 1992, 2001, 2010, and 2019. On average, forests and croplands together occupied 56.2% of the TSLA in 1992–2019, with the rest being wetlands, shrubs, water bodies, urban, and others. Generally, the lower floodplain was mainly covered by forests and wetlands, whereas the upper floodplain was dominated by croplands. For the whole TSLA area, forests covered 2659.1 km^2^ in 1992, which decreased to 1935.8 km^2^ in 2001, 958.5 km^2^ in 2010, but slightly increased to 1018.8 km^2^ in 2019 (about 17%, 12.3%, 6.1%, and 6.5% of the entire area, respectively). As a result, the net forest loss was about 1640.3 km^2^ (with an annual loss rate of −2.3%/year) in 1992–2019. Meanwhile, about 95.9 km^2^ of shrubs were lost (−1.4%/year) during this period. On the other hand, wetlands have considerably increased by 1620 km^2^ over the years. Urban areas have expanded from 11.1 to 63.3 km^2^, croplands have increased by 57.2 km^2^ simultaneously, while water bodies remained roughly unchanged.

**Fig. 3:**
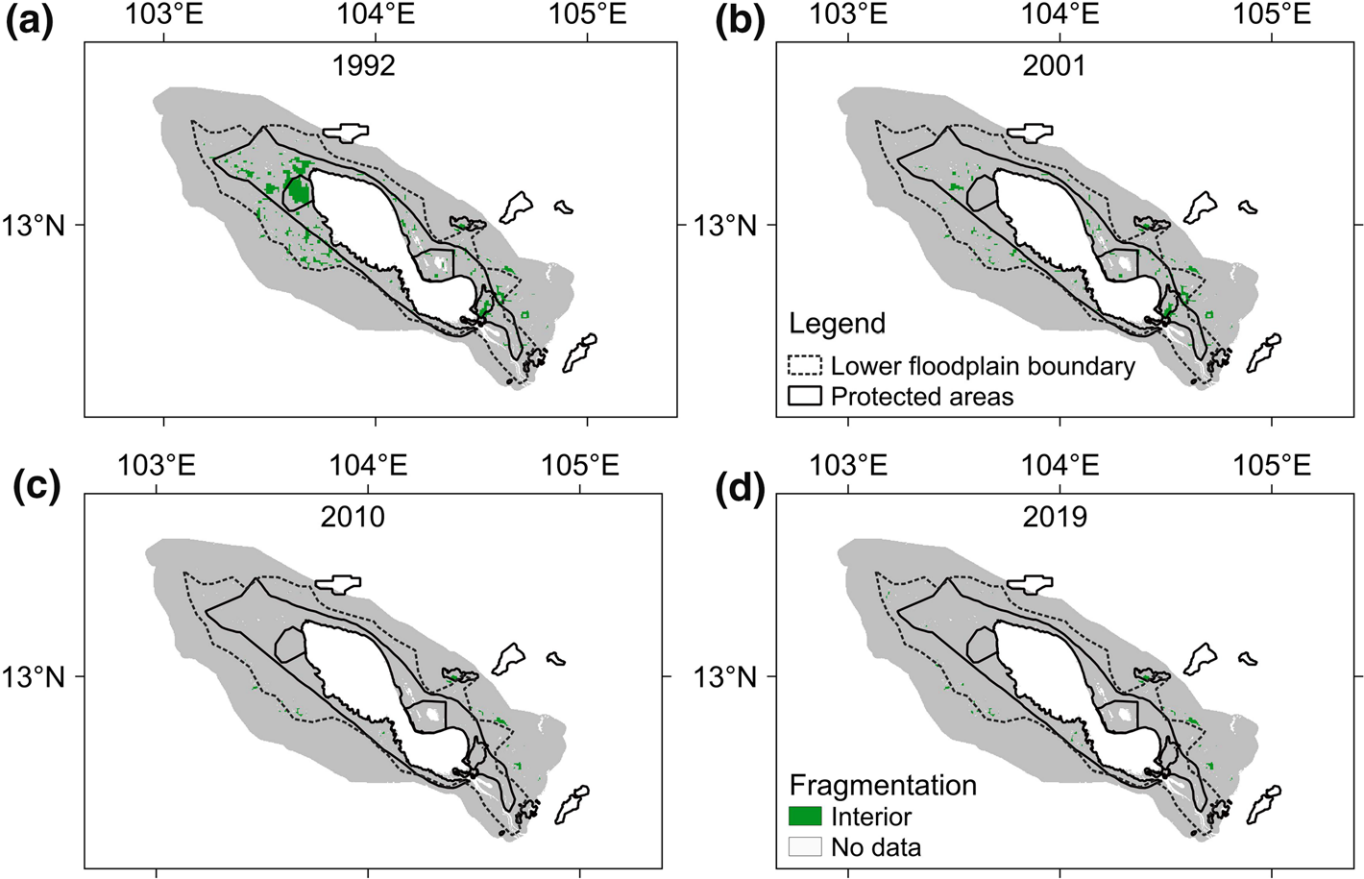
Spatial patterns of forest interior area with 5 × 5 pixel ‘windows’ in the Tonle Sap Lake area in **a** 1992, **b** 2001, **c** 2010, and **d** 2019

Along with the overall trend of forest loss, we also found rapid forest fragmentations since 1992 in the TSLA (Fig. 3a–d). The area of forest interior, representing the least fragmented or intact forest, declined from 500 km^2^ (3.8% of the total area of TSLA) in 1992 to 196.4 km^2^ (1.5%) in 2001, 43.3 km^2^ (0.3%) in 2010, and then slightly recovered to 52.7 km^2^ (0.4%) in 2019. In fact, any forest blocks greater than 1.5 × 1.5 km^2^ had hardly remained since 2010. Such forest fragmentation trends primarily took place in the protected areas at the lower floodplain, where most of the forest interior area were located in 1992. By 2001, however, big patches of forest interior area in the northwest had gone. This fragmentation trend of forest patches had continued until 2010. Similar forest fragmentation patterns can also be revealed by applying other ‘windows’ sizes (i.e., 3 × 3 and 7 × 7 pixels) Fig. S1). Overall, although the TSLA was declared a protected area in 2001, rapid forest fragmentations continued until 2010.

### Forest loss during 1992–2019

**Fig. 4:**
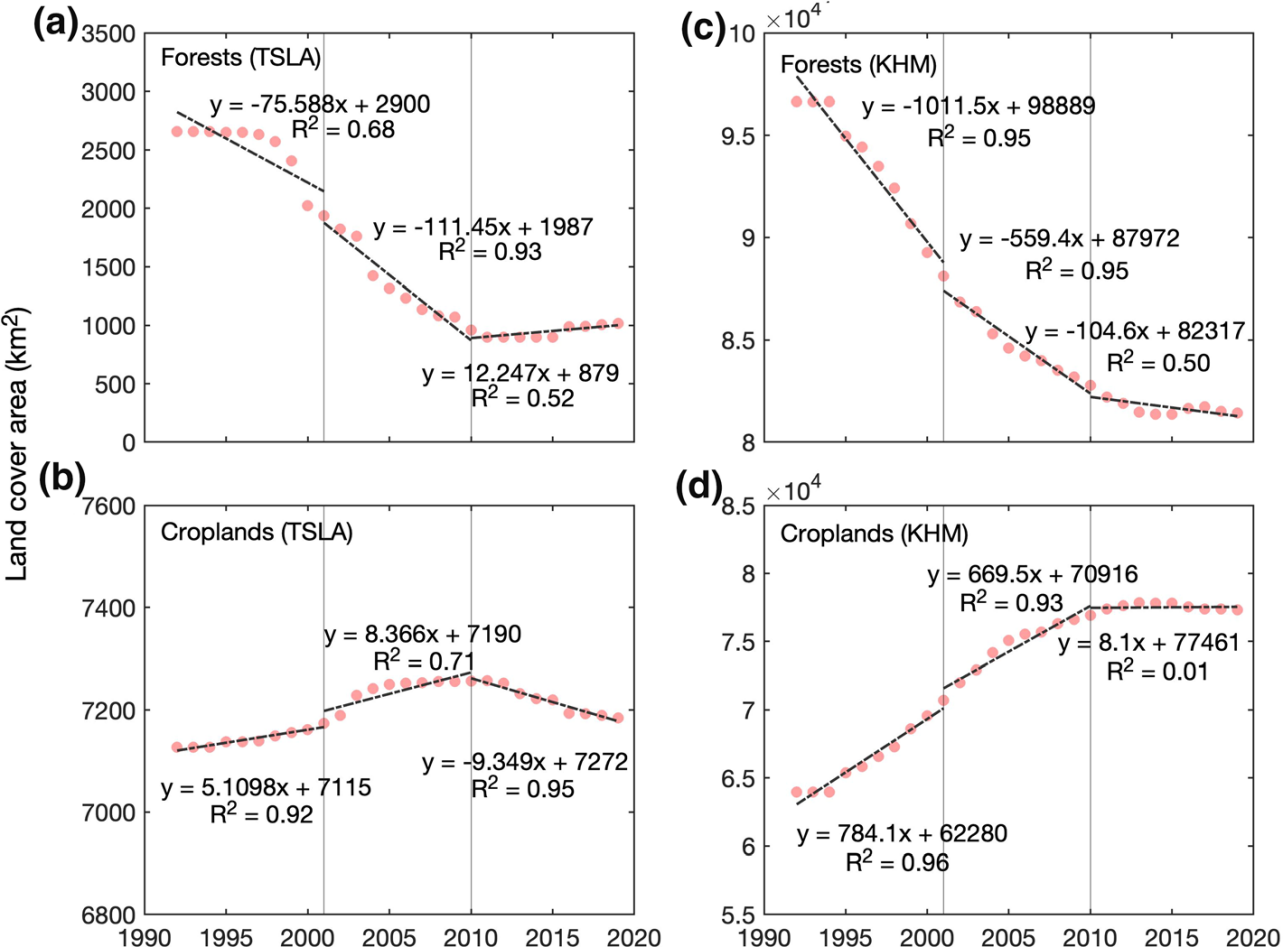
Annual land cover in (**a** and **b)** the Tonle Sap Lake area and (**c** and **d**) and Cambodia: **a**, **c** forests and **b**, **d** croplands. Combined, forests and croplands accounted for about 56.2% (90.6%) of the Tonle Sap Lake area (Cambodia) in 1992–2019. The vertical lines mark the years of 2001 and 2010, and the dashed lines are linear regression lines for 1992–2001, 2001–2010, and 2010–2019, respectively. The regression equations are displayed close to the regression lines

Figure 4 and Table 2 show annual areas of two major land cover classes, forests and croplands, in the TSLA and in Cambodia during 1992–2019. Results again confirmed the continuous forest loss in the TSLA during 2001–2010. In fact, the annual forest loss rates of the TSLA became even more severe from −3.0%/year in 1992–2001 to −5.6%/year in 2001–2010. After 2010, we observed a slight forest regain at the rate of 0.7%/year. As a comparison, Cambodia's annual forest loss rates were −1.0%/year and −0.7%/year in 1992–2001 and 2001–2010, respectively, which were steady losses but much lower (by percentage) than that of the TSLA. After 2010, the total forest area in Cambodia further decreased at a much lower rate of −0.2%/year.

**Table 2:** Annual change rate of land cover types in three sub-periods: 1992–2001, 2001–2010, and 2010–2019 (unit: %/year)

Region	Land cover type	Annual rate 1992–2001	Annual rate 2001–2010	Annual rate 2010–2019
TSLA	Forests	−3.0	−5.6	0.7
	Croplands	0.1	0.1	−0.1
KHM	Forests	−1.0	−0.7	−0.2
	Croplands	1.2	1.0	0.1

Croplands in the TSLA, on the other hand, experienced an increase from 1992 to 2011, followed by a slight decrease. The annual change rates of croplands in the TSLA were 0.07%/year, 0.13%/year, and −0.11%/year for 1992–2001, 2001–2010, and 2010–2019, respectively. Compared to the TSLA, Cambodia as a whole experienced a more prominent cropland expansion till the 2010s. The annual change rates of croplands in Cambodia were 1.17%/year, 0.98%/year, and 0.06%/year for 1992–2001, 2001–2010, and 2010–2019, respectively.

**Fig. 5:**
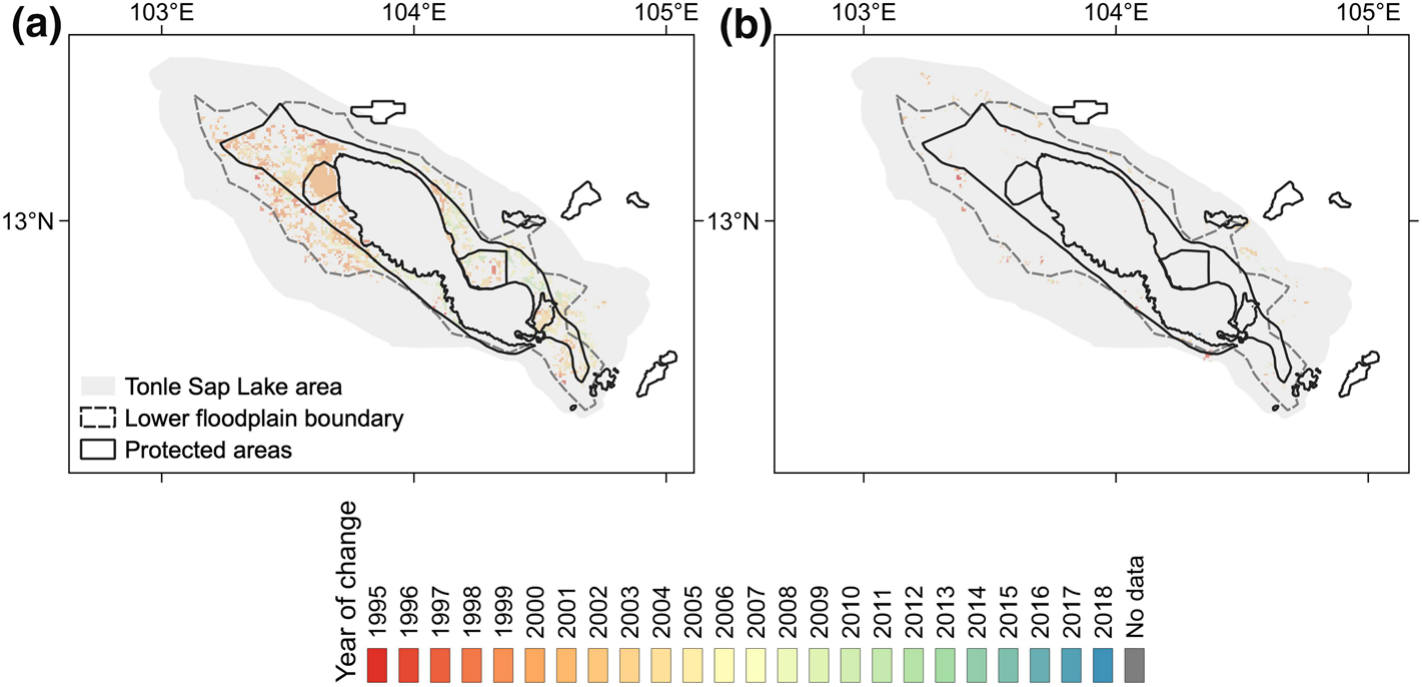
Occurrence of annual land cover change in the Tonle Sap Lake area in 1992–2019: **a** net forest loss, **b** croplands expansion. The year of occurrence is indicated by the color bar below. Forest loss was expanding towards the lake, and new croplands were mainly created at the intersection area of the lower and upper floodplains

The TSLA’s upper and lower floodplains are dominated by different land cover types and subject to different social-economic and management activities. We found that the two subzones possessed diverging land cover changes during the study period. Figure 5a shows that the annual forest loss expanded into the lower floodplain and protected areas. This expansion tendency of forest loss continued after 2001 when the natural protected areas of the UNESCO Biosphere Reserve and the Ramsar site were created, raising concerns about the ecological integrity of these protected areas. Since 2010, the expansion rate has declined. At the same time, areas converted to croplands were primarily located at the intersection area of the lower and upper floodplains (Fig. 5b). These new croplands were mainly converted from shrubs.

### Conversion flow of LUCC during 1992–2019

**Fig. 6:**
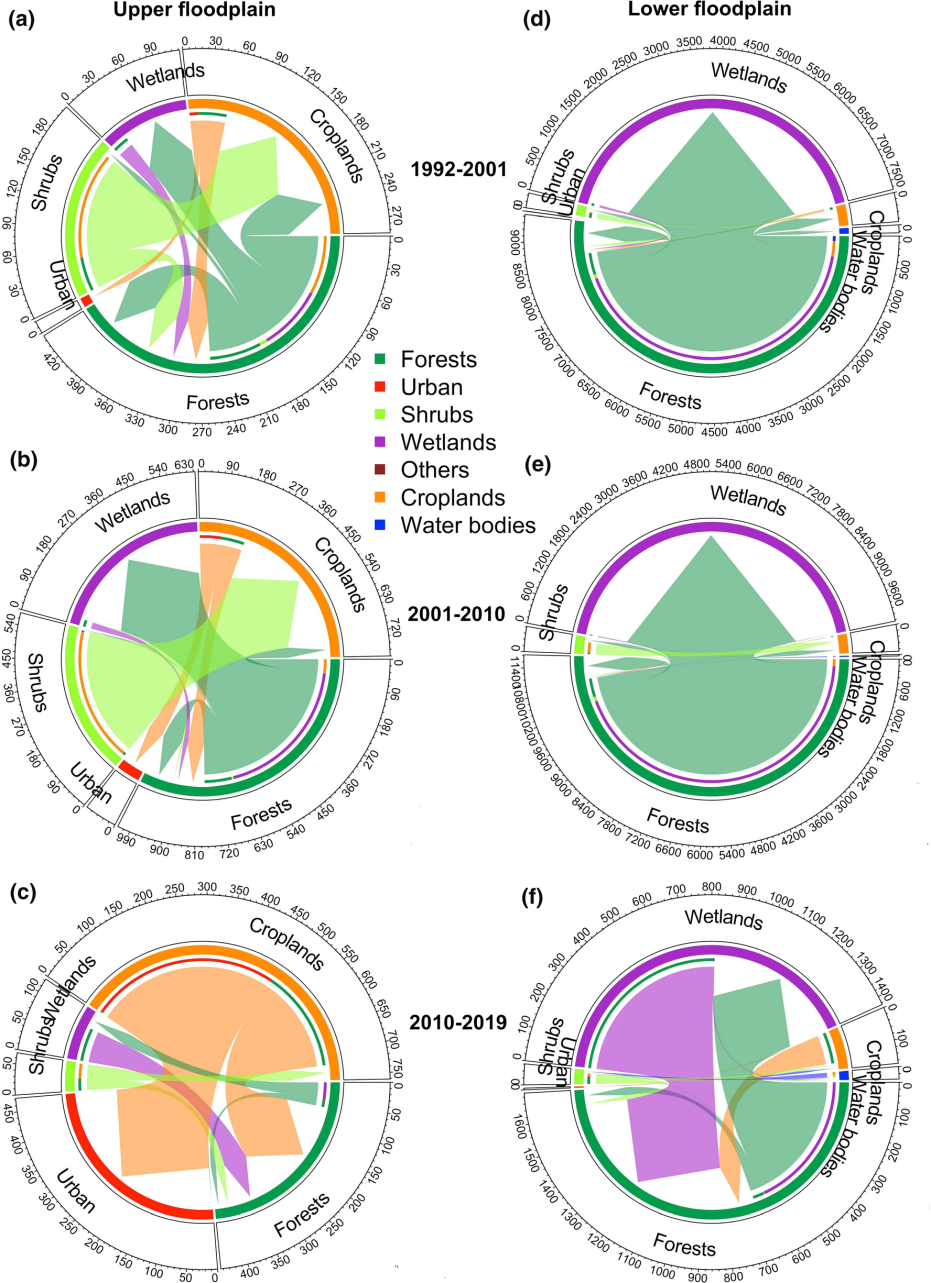
Land cover conversion flow in the Tonle Sap Lake area for **a**, **d** 1992–2001, **b**, **e** 2001–2010, and **c**, **f** 2010–2019: **a**–**c** in the upper floodplain, and **d**–**f** lower floodplain. The color of the section represents land cover types. The size of each colored section represents the proportion of areas of the land cover types, and the number on the axis represents the quantify of land cover types. The arrow indicates the direction of the land cover conversion. For example, the dark green color represents forests, and the direction of the dark green arrow points at the yellow croplands, indicating the conversion from forests to croplands

Conversion flows among the land cover types at the upper and lower floodplains were used to disclose the land conversion processes in the TSLA (Fig. 6). In terms of the land conversions in the whole TSLA between 1992 and 2001 (Fig. S2, and Tables S1–S3), the most extensive land-use conversion was from forests to wetlands, followed by conversions from forests to cropland and shrubs to croplands. Statistically, 91.9% of the forest loss was converted to wetlands (691.1 km^2^), 5.4% of the forest loss was directly converted to croplands (40.5 km^2^), and 54.7% of the shrub loss was converted to croplands (16.1 km^2^). Land conversions in the whole TSLA between 2001 and 2010 presented a similar pattern: 91.8% and 2.3% of the forests were lost to wetlands (960.5 km^2^) and croplands (24.4 km^2^), respectively; and 84.1% of shrubs were converted to croplands (76.1 km^2^). Between 2010 and 2019, land conversions showed very different patterns and were dominated by some forests regain (60.3 km^2^). During this sub-period, 5.6% of forests were still lost to wetlands (59 km^2^), while less than 1 km^2^ of forests were lost to other land cover types. However, more forests were reclaimed from shrubs (5.2 km^2^), wetlands (79.9 km^2^), and croplands (34.9 km^2^). In addition, the growth of urban area was most prominent (42.9 km^2^) since 2010, mostly converted from croplands.

The upper and lower floodplains showed different land cover conversion patterns during 1992–2019. In the upper floodplain, croplands played a significant role among the conversion flows in the three sub-periods (Fig. 6a-c, and Tables S4–S6). For 1992–2001, the most extensive land conversion was from shrubs to croplands (13.5 km^2^). And about three times more shrubs (49.4 km^2^) were converted into croplands in 2001–2010 than in 1992–2001. This shrub-to-cropland conversion was reduced to 3.2 km^2^ in 2010–2019. In addition, urban area expansion mainly occurred in the upper floodplain, primarily from croplands. Temporally speaking, the urban area only expanded by 1 km^2^ between 1992 and 2001; this value increased to 8.0 km^2^ and then surged to 42.3 km^2^ during the latter two sub-periods. In the lower floodplain, forest-to-wetland was the dominant land conversion type (Fig. 6d–f, and Tables S7–S9). About 682.8, 906.3, and 55.3 km^2^ of forests were transformed into wetlands in 1992–2001, 2001–2010, and 2010–2019, respectively. Another 33, 19.1, and 0.2 km^2^ of forests were converted to croplands in the three sub-periods, respectively. And 2.6 km^2^ shrubs were changed to croplands in 1992–2001; this value rose to 26.7 km^2^ in 2001–2010, and dropped to 1.4 km^2^ in 2010–2019.

Comparisons of land cover conversions across the three sub-periods revealed similar but larger forest loss-related conversions in 2001–2010 than in 1992–2001. This extensive forests loss was primarily caused by farming expansion, forest clearing, and forest product collecting during 1992 and 2010.This forest loss-related conversion was significantly reduced after 2010. Overall, the land-use change was intensified after 2001 and the establishment of the protected areas did not curb the trend of forest loss in the TSLA until 2010. On the other hand, although the apparent forest loss was reduced and there had some net gain of forests after 2010, hardly any pristine forests have been left in the TSLA.

Examining the annual time series of forest loss and land conversion flows in the TSLA for 1992–2019, we also found that the amount of forest loss steadily increased from 1992, peaked in 1999 and 2003, and decreased afterward (Fig. S3a). There has been almost no forest loss since 2011, accompanied by some forest regain. Since the forest-to-wetland conversion was the dominant flow, the annual change of forests to wetlands showed a similar temporal pattern as that of forest loss (Fig. S3b). Before 2001, the annual rate of forest-to-cropland conversion increased from 1992 and reached a peak in 2000. Since 2001, this type of land conversion continued to decrease and was nearly zero after 2011 (Fig. S3c). Annual changes of shrubs to croplands showed a monotonous peak in 2002 (Fig. S3d). Overall, the results indicated an increase in forest loss before 2001, which stayed relatively stable during 2001–2010 and reached close to zero after 2010.

## Discussion

### Recent forest loss in the TSLA and Cambodia

Our results indicate a severe net forest loss in the TSLA during 1992–2019 (1640.3 km^2^). This forest loss was close to the result of Lohani et al. (2020), who estimated a total loss of forests at 1944 km^2^ during 1993–2017 with a slightly different Tonle Sap area boundary. Substantial forest loss also existed at the national scale of Cambodia. Using similar methods for Cambodia, we found that Cambodia lost 15,218.2 km^2^ of the forest during 1992–2019, with an annual forest loss rate of 0.6%, which is also close to the 17,150 km^2^ of forest loss in Cambodia during 1993–2017 by Lohani et al. (2020). Most of the forest loss in Cambodia occurred in the northeastern area, driven by the economic land concessions (Davis et al. 2015; Forest Trends 2015; Grogan et al. 2019). Also aligning with Lohani et al. (2020), we found that the TSLA had a higher forest loss rate compared to the national forest loss, even though there are no issued economic land concessions in the region (see Fig. 1). Previous studies have suggested substantial forest loss since the 1960s in the TSLA, which was worse than other areas of Cambodia (Senevirathne et al. 2010). For example, the Tonle Sap Lake watershed's flooded forest was 6140 km^2^ in the 1960s, but it was lost to 3620 km^2^ in 1991 (ADB 2005).

From 1995, rapid forest loss mainly occurred at the lower floodplain, likely because deforestation had already wiped out most forests in the upper floodplain (see Fig. 5). An earlier study indicated that the deforestation frontier has shifted from the lowland to the highland in continental Southeast Asia (Zeng et al. 2018). Here, we show a similar deforestation frontier expansion towards the Tonle Sap Lake. In other words, both studies highlight alarming trends of recent deforestation into areas that are traditionally regarded as less suitable for agriculture and human inhabitation. Deforestation also created severe forest fragmentation in the TSLA (see Fig. 3). In fact, little of the original forest cover remains pristine at the floodplain of the TSLA (Parolin and Wittmann 2010). The annual forest loss rate became greater in 2001–2010 (−5.6%/year) than in 1992–2001 (−3.0%/year). More surprisingly, the forest loss rate in the TSLA was much higher than the nation’s average during 1992–2001 and 2001–2010, despite its essential role in biodiversity conservation (Campbell et al. 2006; Lamberts 2006; Ziv et al. 2012;). However, this high forest loss rate in the TSLA has been stopped since 2010 and a slight forest regain from wetlands, croplands, and shrubs were detected. Meanwhile, the national forest loss rate has also been reduced to −0.2%/year during 2010–2019. Therefore, the establishment of protected areas in the Tonle Sap Lake did not stop forest loss in the TSLA immediately, but with a decadal delayed effect. The stop of forest loss and even a slight forest reclamation after 2010 may be due to combined effects of national forest management reforms, including the Forest Sector Policy Statement and Forestry Law in 2002 and the Protected Area Law in 2008 (McKenney and Tola 2002; MOWRAM et al. 2013; Forest Trends 2015; RECOFTC 2018). Nevertheless, we should bear in mind that the restoration of forested lands even after 2010 was still almost negligible in size and the forest area by 2019 was still much smaller compared to that in the twentieth century.

### Drivers of recent forest loss

Statistics of the fishery and forestry activities in the Tonle Sap region reveal an increase in the number of households engaged in forestry and hunting activities in 2009–2019/2020 (Table 3). Owing to the shrinking flood pulse and decreasing fishing productivities, households with fishery may change to other activities such as agriculture and forestry, which may lead to the increasing number of households engaged in forestry and hunting activities. Such trends could put additional pressure on the farming and ecosystems of Tonle Sap Lake. Furthermore, 90% of Cambodians relied on fuelwood as the primary energy source for cooking in 1998, which has decreased to 83.6% in 2008, and 60.9% in 2019 (NIS 2020b). Driven by the consumption requirements, firewood collection and wood for charcoal dominate among the activities of forestry and hunting in the Tonle Sap, comprising ~ 40.3% of the activities during 2009–2019/2020. However, these two activities have decreased from 42.7% in 2009 to 38.4% in 2019/2020. In addition, sawing logs also presented a decreasing trend from 2.4% to 0.8% simultaneously, which may be due to substantial forest loss leaving not much forest left for forestry and hunting activities.

**Table 3:** Statistics of the fishery and forestry activities in the Tonle Sap region between 2009 and 2019/2020

	Year	2009	2010	2011	2012	2013	2014	2015	2016	2017	2019/2020
Tonle Sap Region	All household (Thousand)	889	888		949	939	998	1033	999	1026	1050
	Number of households with fishing activities by zone (Thousand)	539	532		611	550	509	554	493	447	465
	Number of households with forestry and hunting activities by zone (Thousand)	714	651		736	713	697	785	751	763	730
	Sawing logs (%)	2.4	0.3		2.6	0.7	0.9	0.5	0.7	1.4	0.8
	Firewood (%)	40.5	36.2		37.2	42.1	39.6	38.1	38.9	39.1	37.5
	Wood for charcoal (%)	2.3	2.0		2.1	2.1	0.9	2.1	0.3	0.9	0.9
Cambodia	Sawing logs (%)	1.7	0.6		2.4	1.1	1.3	1.9	0.8	1.1	1.0
	Firewood (%)	43.4	38.7		37.5	42.2	43.5	37.3	39.6	40.6	37.3
	Wood for charcoal (%)	1.0	1.1		2.0	0.9	0.7	2.1	0.6	0.7	0.8

The number of households with fishing, forestry, and hunting activities (1000 households); number of forestry and hunting activities in the Tonle Sap region and in Cambodia (%)

On the national scale, Cambodia also possesses similar patterns of forestry and hunting activities, showing decreasing trends of firewood collection. However, the quantity of firewood collection increased in Cambodia and Tonle Sap region until 2017. Most commercial logging in the catchment was illegal, and illegal logging has destructively eroded the forest cover (McKenney and Tola 2002; Varis and Keskinen 2003; ADB 2005). Overall, firewood collection and logging are among the common causes of forest loss in Cambodia and TSLA until the 2010s (Senevirathne et al. 2010; Grogan et al. 2015).

Weak forest governance may have failed to stop forest loss and deteriorated the forest policy reforms coinciding with the prevailing illegal logging and unsustainable forest product harvesting (Forest Trends 2015; RECOFTC 2018). To revert the trend of rapid deforestation, the Cambodian government launched forest management reforms through the Forest Sector Policy Statement in 2002 and enacted the Forestry Law in 2002 and the Protected Area Law in 2008 (McKenney and Tola 2002; MOWRAM et al. 2013; Forest Trends 2015; RECOFTC 2018). Efforts have also been put to improve the forest governance policies, including the Environment and Natural Resource Code (RECOFTC 2018). For basin-wide management, three organizations were established: the Tonle Sap Biosphere Reserve (TSBR) and its secretariat (driven by UNESCO and the Cambodian government), the Tonle Sap Basin Management Organization (TSBMO, driven by the Asian Development Bank), and the Tonle Sap Basin Authority (TSBA, now the Tonle Sap Authority, run by the Cambodian government) (Sithirith 2015). However, having different views and interests towards the basin development, the three Tonle Sap management organizations resulted in weak institutional coordination and overlapping mandates (Keskinen 2010; Sithirith 2015). Moreover, they are simply coordinating institutes without effective authorities (Keskinen 2010). Evidence of other protected areas in Cambodia has also experienced substantial forest loss even after 2001 (Collins and Mitchard 2017). Our results showed that the TSLA had experienced continuous forest loss during 2001–2010. Although the trend has been paused since 2010, only a few small forest patches were left in the floodplain. Therefore, the government’s attempt to regulate forest exploitation with forest reforms so far has had limited success at the Tonle Sap Lake ecoregion.

Hydropower development is estimated to cause water levels to fall in the wet season and rise in the dry season (Kummu and Sarkkula 2008; Arias et al. 2012; Keskinen et al. 2015). This flood pulse change can be one of the drivers for forest loss (Lin and Qi 2017). The annual flood pulse in the Tonle Sap Lake influences human use of vegetation, ranging from wood product harvesting to vegetation clearance (Arias et al. 2013; Salmivaara et al. 2016). For instance, vegetation clearance happened in areas that experienced a shorter flood period in particular, while selected wood harvesting often occurred in areas with a more prolonged flood (Arias et al. 2013). Shrinking flood pulse from the late 1990s (Chen et al. 2021) could reduce seasonally flooded habitat and influence human use of forest and other vegetation (Arias et al. 2012, 2013).

Intensified agriculture activity is also a driver of forest loss (Song et al. 2011). Rice cultivation has induced 28% of the detected Tonle Sap grassland cover loss in 2005–2007 (Gray et al. 2009). Expanding new cropland was found at the lower and upper floodplains intersection between 1992 and 2019. Escalating agriculture could partly be pushed by the diminishing flood pulse because it determines the floodplain rice production, delimited in the floodplain's outer part with irregular flooding (Arias et al. 2012; Kleinhenz et al. 2013). Overall, intensified agriculture activity and hydropower development could reduce the buffer to natural habitats and increase the risk of further forest loss.

### Socioeconomic challenges

TSLA has witnessed significant demographic and socioeconomic changes in recent decades (Arias et al. 2013; Keskinen et al. 2015). The occupation of people living in the TSLA is highly concentrated on fishing (mainly lower floodplain) and agriculture (upper floodplain), suggesting a high dependency on natural resources (Keskinen 2006; Salmivaara et al. 2016). Continuous forest loss deteriorates the historical “safety net” for the rural poor who have no land, limited livelihood opportunities, and living on collecting forest products for subsistence (McKenney and Tola 2002). Rapid population growth and a large share of young cohorts since the 2000s further added substantial pressures on the TSLA ecosystem (McKenney and Tola 2002; Chadwick et al. 2008; Salmivaara et al. 2016). Even though the proportions of the workforce within fishing and agriculture decreased slowly in 1998–2008, there was a significant increase in the number of people in these sectors (Salmivaara et al. 2016). As population growth has driven the rapid cropland expansion (Keskinen et al. 2015), Song et al. (2011) argued that extending productive areas has not substantially increased farmers’ income nor improved their livelihood. Indeed, the level of livelihoods has seen depletion, accompanied by the rising population that exacerbates people’s vulnerability to environmental changes in the TSLA (Keskinen 2006). To decouple the economic development from increased use of natural resources, enhancing the diversification of the economy (e.g., developing tertiary industry) could increase the resilience of people’s livelihood. Regional land-use strategies should accommodate both short- and long-term needs, balance ecosystem services, and consider the demographic transition, expected environmental impacts of hydropower development, and climate change (Foley et al. 2005; Salmivaara et al. 2016). Furthermore, properly managing policy implementation is fundamental to ensure the effective performance of the policy.

### Limitations

The major uncertainties that exist in the study come from the land cover product we used, the ESA CCI land cover product (300 m, 1992–2019). Many satellite-based land cover data are available, including the MODIS MCD12Q1 (500 m, 2001-present) (Sulla-Menashe et al. 2019), Global Land Cover 2000 (1000 m, 2000) (Bartholomé and Belward 2005), GlobeLand30 (30 m, 2000, 2010, 2020) (Chen et al. 2014) and Global Forest Change (30 m, 2000–2019) (Hansen et al. 2013), and the Lower Mekong Regional Land Cover Monitoring System (30 m, 2001–2017) (Potapov et al. 2019). To estimate the change of forest cover in the Tonle Sap Lake and evaluate the efficiency of the protected area established in 2001, land cover products without data available before 2000 were not considered. Therefore, ESA CCI is the one that meets the requirements, and it offers all the major land cover classes allowing land cover conversion analysis to understand better the major land conversion associated with forest loss. However, due to the relatively coarse horizontal resolution of ESA CCI (300 m), it may underestimate the small patches of forest and other land covers. In this study, we have compared the annual forest cover estimated from ESA CCI with the inventory data collected from various sources (i.e., FAO, Cambodian government reports) (Table 4). Results showed that ESA CCI’s land cover estimate is close to these sources, proving the reliability of this land cover product in Cambodia.

**Table 4:** Statistics of forest cover in Cambodia from multiple forest inventory (unit: km^2^)

Year	a	b	c	d	e
1992	113 588	108 919			96 642
1996		106 719			94 425
2002			113 924		86 866
2006				108 317	84 223
2010				104 519	82 778
2014				85 182	81 372
2016				81 819	81 647

^a^The Cambodia Land Cover Atlas, published in 1994, with support from FAO/UNDP (Brun 2013)

^b^Forest Cover Monitoring Project (FCMP) 1992/1993–1996/1997 (Brun 2013)

^c^Brief on national forest inventory NFI, Cambodia, 2007 (Piazza and Govil 2007)

^d^Cambodia forest cover 2016 (Ministry of Environment 2018)

^e^Our estimate from ESA CCI land cover product (ESA 2017)

On the other hand, we compared land cover changes in the TSLA and in Cambodia to evaluate the effectiveness of forest management policies, similar to previous studies (e.g., Potapov et al. (2019) and Lohani et al. (2020). Ideally, such comparisons should be made between the TSLA and its immediately neighboring areas that were not protected, i.e., a buffer zone from the TSLA that shares similar environmental and socioeconomic settings with the only exception in protection status. However, since Cambodia’s economic land concessions and protected areas are scattered around the study area, it is not realistic to find such an ideal buffer zone within a given distance threshold for a “fair” comparison (see Fig. 1a). Nonetheless, the comparison results between land cover changes in the TSLA and in Cambodia should be carefully interpreted considering that the different environmental and socioeconomic contexts between the two regions may also contribute to their differences in land cover changes. Other uncertainties may arise from the definition of ‘forest’ in the land cover products. There are about 800 official definitions of the ‘forest,’ and disagreements among the land cover products source deeply from such many different definitions (Sexton et al. 2016). To be more accurate with forest assessment, efforts to refine forest monitoring should be made among academia and the governments.

## Conclusions

The study reveals severe forest loss in the TSLA during 2001–2010, even though forest reforms have been in place since the early 2000s. Specifically, it was found that (i) forests were fragmented with barely any intact forest left from 2010, (ii) forest loss hotspots were at the lower floodplain where protected areas are located, and (iii) notable cropland expansion was mainly located at the intersection area between the lower and upper floodplains. Compared with forest cover change in 1992–2001, evidence shows that the forest reforms did not halt forest loss in the TSLA in 2001–2010. And the forest loss rate was much higher than the Cambodian national rate for 2001–2010. This forest loss in the TSLA has been paused since 2010 with even some forest regain. However, forest recovery since 2010 was almost negligible and the large net forest loss over the entire period (1992–2019) still posed a grand challenge for restoring the ecological integrity of this internationally important protected area. Generally, population growth, firewood collection, and logging are leading causes of forest loss. Meanwhile, intensified agriculture activity in the TSLA and the upstream hydropower development further increases the risk of forest loss by reducing the buffer to natural habitats. Facing the challenge of meeting human demands and maintaining the functional capacity of the ecosystem in the TSLA in the long term, this study offers scientific evidence for understanding the human interference on forests that can be useful for future sustainable forest management to achieve a healthy ecosystem in the TSLA, the rest of Cambodia, and other areas facing similar socioeconomic and forest loss issues.

## Supplementary Information

Below is the link to the electronic supplementary material.

Supplementary file1 (DOCX 1075 kb)
