# Supplementary material for: Large net forest loss in Cambodia’s Tonle Sap Lake protected areas during 1992–2019
Source: Ambio. 2022 Feb 8;51(8):1889–903. doi: 10.1007/s13280-022-01704-4 (PMC9200915; doi:10.1007/s13280-022-01704-4)
Supplement: Supplementary file 1 — Supplementary file1 (DOCX 1075 kb) [file 13280_2022_1704_MOESM1_ESM.pdf]

Ambio

Electronic Supplementary Material

This supplementary material has not been peer reviewed.

Title: **Large net forest loss in Cambodia's Tonle Sap Lake protected areas during 1992-2019**

Authors: Aifang Chen, Anping Chen, Olli Varis, Deliang Chen

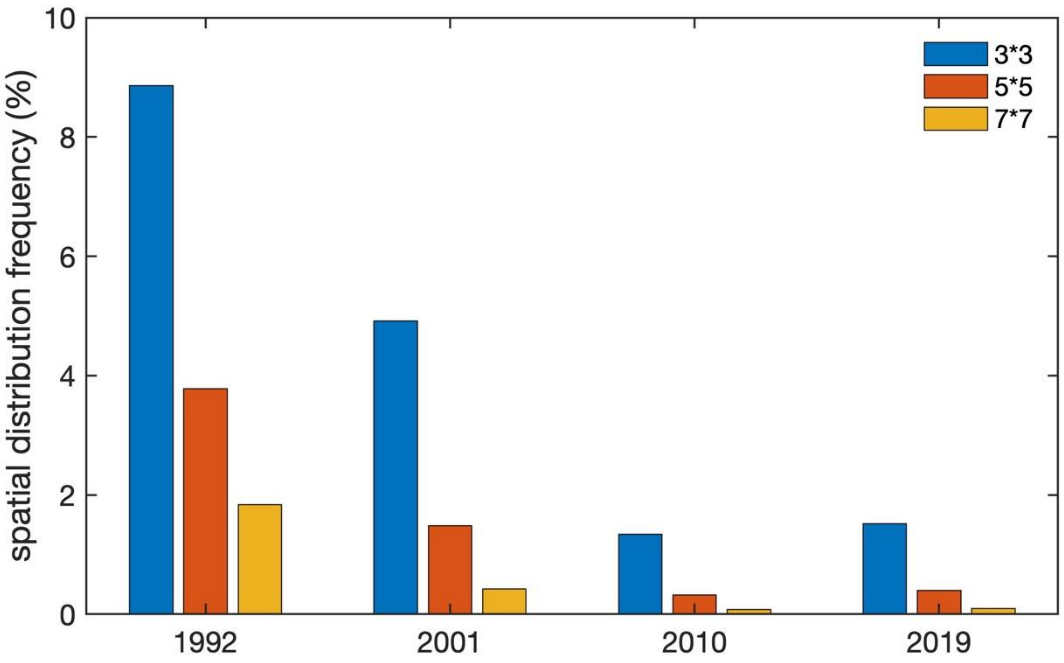

Figure S1 Percentage area of the forest interior area in the Tonle Sap Lake area in 1992, 2001, 2010 and 2019 with various sizes of 'windows' (including, 3×3, 5×5, and 7×7 pixels).

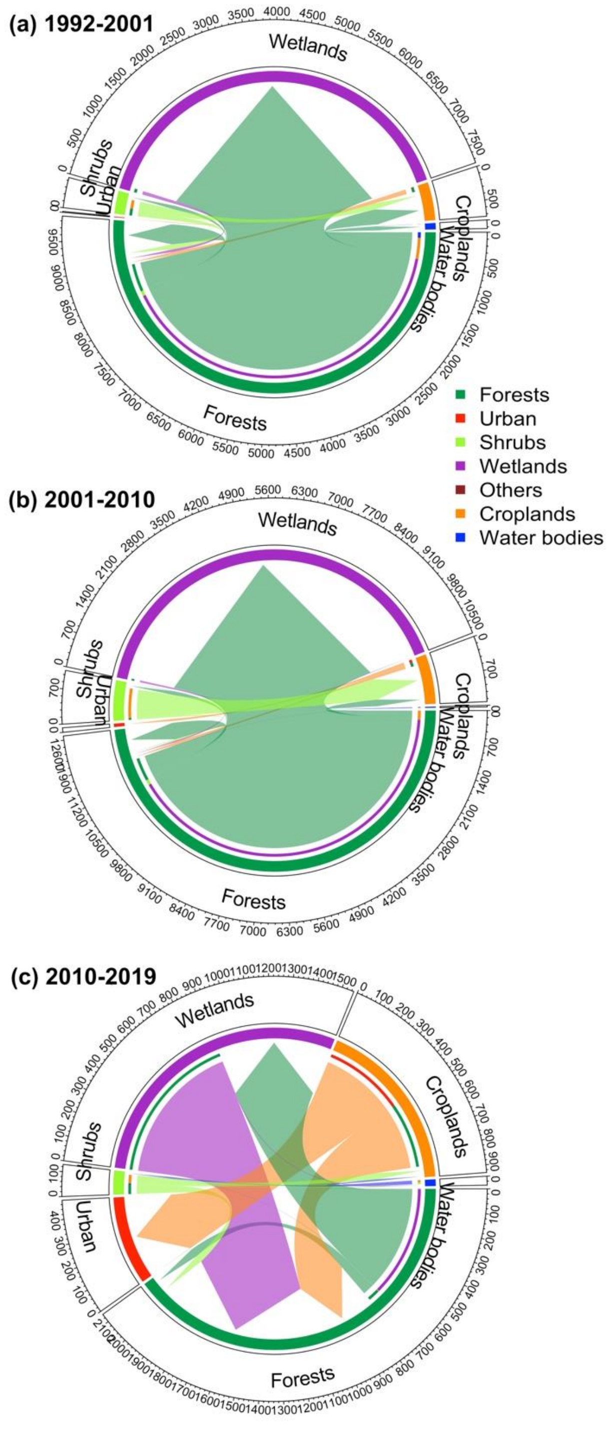

Figure S2 Land cover conversion flow in the whole Tonle Sap Lake area for (a) 1992–2001, (b) 2001–2010 and (c) 2010–2019. The color of the section represents land cover types. The size of each colored section represents the proportion of areas of the land cover types, and the number on the axis represents the quantify of land cover types. The arrow indicates the direction of the land cover conversion. For example, the dark green color represents forests, and the direction of the dark green arrow points at the yellow croplands, indicating the conversion from forests to croplands.

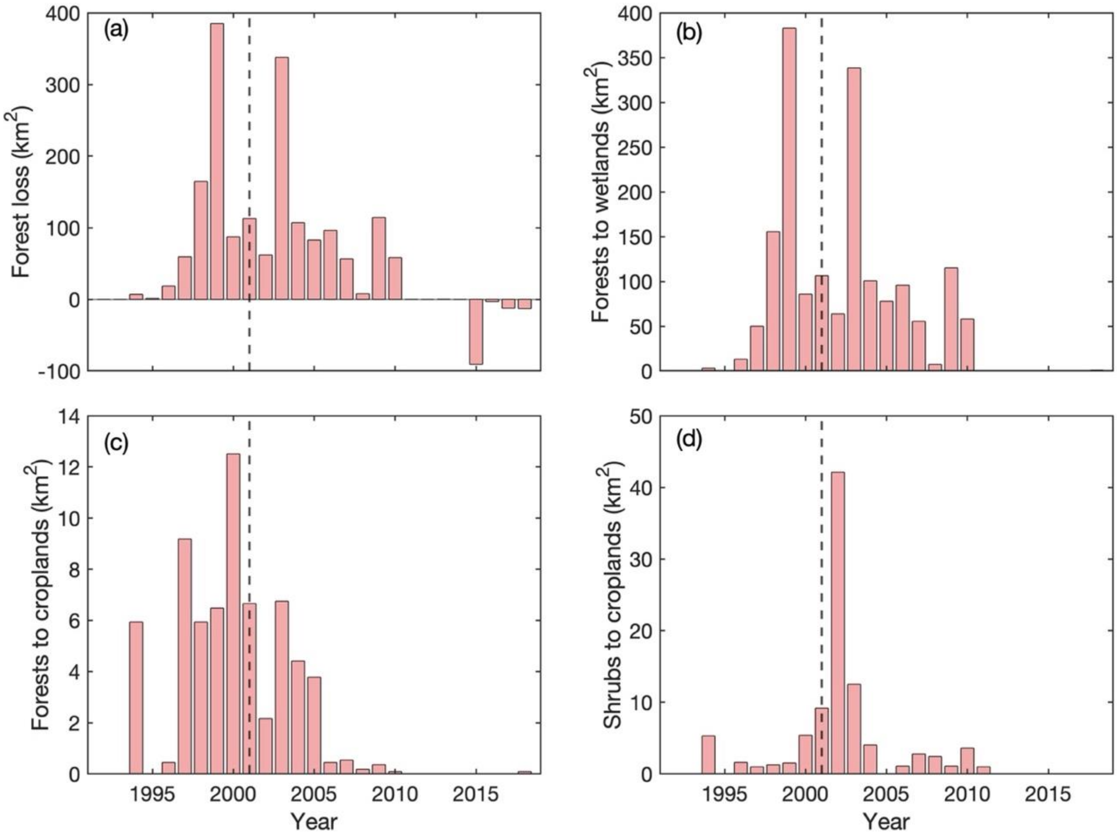

Figure S3 Time series of forest loss and associated land conversion flow in the whole Tonle Sap Lake area for 1992–2019: (a) Forest loss, (b) forests converted to wetlands, (c) forests converted to croplands, and (d) shrubs converted to croplands.

Table S1 Land cover conversion flow among the land classes in the whole TSLA between 1992–2001(unit: km^2)

Classes	Forests	Shrubs	Wetlands	Croplands	Urban	Others	Water bodies
Forests	53.6	9.9	691.1	40.5	0.0	0.0	10.6
Shrubs	13.3	0.3	0.0	16.1	0.0	0.0	0.0
Wetlands	7.1	0.0	0.0	0.0	0.0	0.0	0.1
Croplands	8.4	0.0	0.0	0.0	1.3	0.0	0.0
Urban	0.0	0.0	0.0	0.0	0.0	0.0	0.0
Others	0.0	0.0	0.0	0.0	0.0	0.0	0.0
Water bodies	0.1	0.0	0.3	0.1	0.0	0.0	0.0

Table S2 Land cover conversion flow among the land classes in the whole TSLA between 2001–2010 (unit: km^2)

Classes	Forests	Shrubs	Wetlands	Croplands	Urban	Others	Water bodies
Forests	61.0	13.5	960.5	24.4	0.0	0.0	0.0
Shrubs	5.1	0.0	0.5	76.1	0.0	0.0	0.0
Wetlands	5.0	0.0	0.0	0.0	0.0	0.0	0.0
Croplands	10.3	0.0	0.0	0.0	8.0	0.0	0.0
Urban	0.0	0.0	0.0	0.0	0.0	0.0	0.0
Others	0.0	0.0	0.0	0.0	0.0	0.0	0.0
Water bodies	0.7	0.7	1.3	0.0	0.0	0.0	0.0

Table S3 Land cover conversion flow among the land classes in the whole TSLA between 2010–2019 (unit: km^2)

Classes	Forests	Shrubs	Wetlands	Croplands	Urban	Others	Water bodies
Forests	6.2	0.3	59.0	0.2	0.2	0.0	0.2
Shrubs	5.2	0.0	0.0	4.6	0.0	0.0	0.4
Wetlands	79.9	0.0	0.0	0.0	0.0	0.0	0.5
Croplands	34.9	0.2	0.0	0.0	42.8	0.0	0.1
Urban	0.0	0.0	0.0	0.0	0.0	0.0	0.0
Others	0.0	0.0	0.0	0.0	0.0	0.0	0.0
Water bodies	0.0	1.0	0.0	1.1	0.0	0.0	0.0

Table S4 Land cover conversion flow among the land classes in the TSLA's upper floodplain between 1992–2001 (unit: km^2)

Classes	Forests	Shrubs	Wetlands	Croplands	Urban	Others	Water bodies
Forests	6.6	1.0	8.3	7.5	0.0	0.0	0.0
Shrubs	4.3	0.0	0.0	13.5	0.0	0.0	0.0
Wetlands	2.1	0.0	0.0	0.0	0.0	0.0	0.0
Croplands	3.6	0.0	0.0	0.0	1.1	0.0	0.0
Urban	0.0	0.0	0.0	0.0	0.0	0.0	0.0
Others	0.0	0.0	0.0	0.0	0.0	0.0	0.0
Water bodies	0.0	0.0	0.0	0.0	0.0	0.0	0.0

Table S5 Land cover conversion flow among the land classes in the TSLA's upper floodplain between 2001–2010 (unit: km^2)

Classes	Forests	Shrubs	Wetlands	Croplands	Urban	Others	Water bodies
Forests	10.1	0.7	54.2	5.3	0.0	0.0	0.0
Shrubs	0.9	0.0	0.5	49.4	0.0	0.0	0.0
Wetlands	2.4	0.0	0.0	0.0	0.0	0.0	0.0
Croplands	8.6	0.0	0.0	0.0	8.0	0.0	0.0
Urban	0.0	0.0	0.0	0.0	0.0	0.0	0.0
Others	0.0	0.0	0.0	0.0	0.0	0.0	0.0
Water bodies	0.0	0.0	0.0	0.0	0.0	0.0	0.0

Table S6 Land cover conversion flow among the land classes in the TSLA's upper floodplain between 2010–2019 (unit: km^2)

Classes	Forests	Shrubs	Wetlands	Croplands	Urban	Others	Water bodies
Forests	1.6	0.0	3.7	0.0	0.2	0.0	0.0
Shrubs	2.7	0.0	0.0	3.2	0.0	0.0	0.0
Wetlands	7.4	0.0	0.0	0.0	0.0	0.0	0.0
Croplands	22.8	0.2	0.0	0.0	42.3	0.0	0.0
Urban	0.0	0.0	0.0	0.0	0.0	0.0	0.0
Others	0.0	0.0	0.0	0.0	0.0	0.0	0.0
Water bodies	0.0	0.0	0.0	0.0	0.0	0.0	0.0

Table S7 Land cover conversion flow among the land classes in the TSLA's lower floodplain between 1992–2001 (unit: km^2)

Classes	Forests	Shrubs	Wetlands	Croplands	Urban	Others	Water bodies
Forests	47.1	8.9	682.8	33.0	0.0	0.0	10.6
Shrubs	9.0	0.3	0.0	2.6	0.0	0.0	0.0
Wetlands	5.0	0.0	0.0	0.0	0.0	0.0	0.1
Croplands	4.8	0.0	0.0	0.0	0.2	0.0	0.0
Urban	0.0	0.0	0.0	0.0	0.0	0.0	0.0
Others	0.0	0.0	0.0	0.0	0.0	0.0	0.0
Water bodies	0.1	0.0	0.3	0.1	0.0	0.0	0.0

Table S8 Land cover conversion flow among the land classes in the TSLA's lower floodplain between 2001–2010 (unit: km^2)

Classes	Forests	Shrubs	Wetlands	Croplands	Urban	Others	Water bodies
Forests	50.9	12.8	906.3	19.1	0.0	0.0	0.0
Shrubs	4.2	0.0	0.0	26.7	0.0	0.0	0.0
Wetlands	2.5	0.0	0.0	0.0	0.0	0.0	0.0
Croplands	1.7	0.0	0.0	0.0	0.0	0.0	0.0
Urban	0.0	0.0	0.0	0.0	0.0	0.0	0.0
Others	0.0	0.0	0.0	0.0	0.0	0.0	0.0
Water bodies	0.7	0.7	1.3	0.0	0.0	0.0	0.0

Table S9 Land cover conversion flow among the land classes in the TSLA's lower floodplain between 2010–2019 (unit: km^2)

Classes	Forests	Shrubs	Wetlands	Croplands	Urban	Others	Water bodies
Forests	4.6	0.3	55.3	0.2	0.0	0.0	0.2
Shrubs	2.5	0.0	0.0	1.4	0.0	0.0	0.4
Wetlands	72.5	0.0	0.0	0.0	0.0	0.0	0.5
Croplands	12.2	0.0	0.0	0.0	0.5	0.0	0.1
Urban	0.0	0.0	0.0	0.0	0.0	0.0	0.0
Others	0.0	0.0	0.0	0.0	0.0	0.0	0.0
Water bodies	0.0	1.0	0.0	1.1	0.0	0.0	0.0
